# Mycotoxin binders potential on histological of ovary mice exposed by zearalenone

**DOI:** 10.14202/vetworld.2017.353-357

**Published:** 2017-03-27

**Authors:** Abdul Samik, Erma Safitri

**Affiliations:** 1 Department of Veterinary Reproduction, Faculty of Veterinary Medicine, Universitas Airlangga, Indonesia; 2 Stem Cells Research Division of Institute Tropical Disease, Universitas Airlangga, Indonesia

**Keywords:** corpus luteum, follicles, mycotoxin binders, zearalenone

## Abstract

**Aim:**

This study was conducted to examine the potential of mycotoxin binder in ceasing zearalenone (ZEN) effect on mice reproduction. ZEN mycotoxin can induce reactive oxygen species that may cause damage and cell death. ZEN is estrogenic so that it may affect the reproductive organs failure.

**Materials and Methods:**

Mycotoxin binder administration to female mice exposed to ZEN was aimed to count the number of primary follicles, secondary follicles, tertiary follicles, de Graaf’s follicles, and the corpus luteum (CL). Negative control group (C) was not exposed to ZEN and without the administration of mycotoxin binders, while positive control group (C+) was exposed to 0.1 mg/mouse/day ZEN and without the provision of mycotoxin binders. Treatment groups (T1, T2, T3) were exposed to 0.1 mg/mouse/day ZEN and mycotoxin binders 0.5; 1; 2 mg/BW/day.

**Results:**

ZEN and mycotoxin binders administration was conducted for 10 days. The number of primary follicles, secondary, tertiary, de Graaf’s follicles and CL in negative control (C−) was 14.2±1.36, 11.2±0.28, 6.5±0.53, 7.5±0.74, and 2.3±0.35. The number in positive control (C+) group was as follows 7.1±0.12, 3.7±1.17, 3.8±1.21, 1.5±0.62, and 2.3±0.34. Results in treatment 1 (T1) were as follows 6.2±0.16, 5.2±0.16, 3.6±0.16, 2.6±0.19, and 2.6±0.10; in treatment 2 (T2) 7.8±0.28, 5.8±0.53, 3.7±0.26, 2.7±0.26, and 2.5±0.10; and in treatment 3 (T3) 8.4±0.34, 8.4±0.34, 4.6±0.34, 4.5±1.01, and 3.4±0.23.

**Conclusion:**

The number of follicles and CL more in line with increasing doses of mycotoxin binders. Required more than 2 mg/mouse/day mycotoxin binders to inhibit the effects of ZEN so that its can maintain the number of primary follicle, secondary follicle, tertiary follicle, the de Graaf’s follicle, and the number of CL in the ovary of ZEN-exposed female mice (*Mus musculus*).

## Introduction

Folliculogenesis in an animal species is affecting the development of oocytes (egg cells). The number of follicles during estrus cycle is influenced by factors such as animal species, reproductive phase, circumstances, age, mother, and genetic. Folliculogenesis includes changes in the size and number of granulosa cells (GCs), theca cell growth, egg cells position which is surrounded by cumulus oophorus cells, and increased volume of follicular cavity fluid [1]. Effects of estrogen on ovarian organ may increase the growth of granulosa and cumulus oophorus cells in reaching egg cells maturation [2].

At molecular level, hyperestrogenism conditions caused by zearalenone (ZEN) from the fungus *Fusarium graminearum* may lead to oxidative stress and induce apoptosis through intrinsic pathway in the mitochondria. The intrinsic pathway involves mitochondrial function affected by oxidative stress by releasing protein and activating caspase release into the cytosol. Caspase is a protease with a capacity to break down proteins. Oxidative stress makes cytochrome be released out of the mitochondria and will bind apoptotic protease activating factor 1 and procaspase 9 to activate Caspase 9 [3]. Caspase 9, which acts as the apoptosis initiator, will be dimerized and trigger a feedback by inhibiting Bcl-2 release and binds procaspase 3 to activate Caspase 3. Caspase 3 acts as executor, helping endonuclease and cytoplasmic protease activation that may fragment the nuclear DNA and degrade protein cytosol. The final result in fragmentation process is the formation of apoptotic bodies containing intracellular organelles and express phosphatidylserine that will trigger phagocytosis [3,4].

Mycotoxin binders act effectively in binding mycotoxins in the feed. Mycotoxin binders contain material that with high potential to be absorbed, including aluminosilicate activated carbon, cellulose, polysaccharides, peptidoglycan, and synthetic polymers such as cholestyramine, polyvinylpyrrolidone, and their derivatives [5]. The action mechanism of mycotoxin binders by eliminating methyl groups on ZEN’s chemical structure [6]. The basic materials of mycotoxin binders are in the form of charcoal compound, biotin, thiamine, aluminosilicate, and vitamins C and E which are antioxidants to decrease the effects of exposure to mycotoxins residues before being metabolized by the body.

The objective of this study was to determine the effect of mycotoxin binders on the number of primordial follicle cells, primary follicle, secondary follicle, tertiary follicle, de Graaf’s follicle, and the corpus luteum (CL) in mice (*Mus musculus*) exposed to ZEN. This mycotoxin binders potential may be a solution to overcome problems of estrus cycle disorders due to ZEN which is produced by the fungus *F. graminearum* and to improve livestock productivity by controlling mycotoxins from the feed.

## Materials and Methods

### Ethical approval

This study was approved by ethical committee vide Ethical Clearance No: 309-KE Animal Care and use Committee, Faculty of Veterinary Medicine Airlangga University.

### Experimental

This study was an experimental laboratory using a completely randomized design. Data were statistically analyzed using SPSS 15 for Windows XP with the level of significance 0.05 (p=0.05) and the confidence level 99% (α=0.01). Steps of comparative hypothesis tests are as follows: Kolmogorov–Smirnov for normality test when the data normal distribution than continued with one-way ANOVA test and if significant difference (p<0.05) was found; the analysis was followed with Fisher’s least significant difference test. A total of 20 female mice (*M. musculus*) from animal treatment laboratory, Faculty of Veterinary Medicine Airlangga University, were used in this study. The laboratory animals used in this study were healthy female mice, 8-10 week-old and each 20-30 g weight. Healthy condition was determined by their active movement. Mice kept in an individual plastic cage in laboratory for Experimental Animal of Veterinary Medicine, Faculty of Universitas Airlangga with adequate ventilation. They were divided into five treatments in which each treatment was subjected to 4 replications as follows: Negative control group (C), not exposed to ZEN (Biotech Co. Ltd., Hangzhou, Zhejiang, China) and without being administered with mycotoxin binders; positive control group (C+), exposed to ZEN dose of 0.1 mg/mouse/day without receiving mycotoxin binders; treatment group 1 (T1) exposed to ZEN dose of 0.1 mg/mouse/day and the provision of mycotoxin binders (Impextraco Ltd, Bangkok, Thailand) 0.5 mg/mouse/day; treatment group 2 (T2) exposed to ZEN dose of 0.1 mg/mouse/day and receiving mycotoxin binders dose of 1 mg/mouse/day; the treatment group 3 (T3) exposed to ZEN dose of 0.1 mg/mouse/day and mycotoxin binders in a dose of 2 mg/mouse/day. Variables were observed in this study and were the number of primary follicle, secondary follicle, tertiary follicle, de Graaf’s follicle, and the CL. Selection of mycotoxin binders concentration was based on previous research [5].

### Procedures for mycotoxin binders and ZEN administration

Mycotoxin binders were administered orally to the female mice in doses of 0 mg/mouse/day, 0.5 mg/mouse/day, 1 mg/mouse/day, 2 mg/mouse/day, and in each treatment ZEN was added in a dose of 0.1 mg/mouse/day. The administration was done orally with a gastric sonde needle (Kent Scientific Co, Connecticut, USA) and repeatedly done until the 10^th^ day. Gastric sonde needle in this research is instrument model of feeding needle for animal laboratory, Connecticut, USA.

### Animal surgical procedures and sampling

After the dislocation of the cervical Os, surgery was performed on day 15. Disinfection was done with 70% alcohol followed by quick surgery to take out the ovaries. The ovaries were extracted and separated from the surrounding tissue, then stored in buoin solution with 75 ml saturated picric acid, 25 ml 40% formalin, and 5 ml glacial acetic acid for making preparations for immunohistochemistry and histology [7].

### Observations of ovarian histology with hematoxylin-eosin staining [7]

Mice ovaries were fixed with buoin solution. The process was followed with hematoxylin and eosin staining. The staining was begun with soaking the object glass into xylol I and xylol II, each for 2 min, followed by immersion in 95%, 90%, 80%, 70% and 50% absolute (100%) alcohol respectively for 2 min. Then, the glass objects were put into hematoxylin staining for 7 min and washed with running water to remove the unabsorbed excess dye. Object glasses were re-immersed in eosin staining for 3 min and washed again with distilled water. The preparations were then soaked in 50%, 70%, 85%, 90%, 100%, 100%, alcohol, xylol I, and xylol II respectively for 2 min. Chemical was used in this study from Biotech Co. Ltd., Hangzhou, Zhejiang, China.

## Results

Ovarian histological feature reading was performed on all preparations microscopically using quantitative methods for the number of the primary follicle, secondary follicle, tertiary follicle, de Graaf’s follicle, and the CL. Ovarian Caspase 9 expression reading was performed on all preparations microscopically using scoring method [7]. The results of counting scoring obtained were subsequently averaged and then processed using SPSS 20 for windows. The means and standard deviations of the observations of the number of follicles, the CL and Caspase 9 are presented in Table-1.

**Table-1:** Mean and standard deviation of observed follicle numbers and CL.

Treatments	Follicular count (X±SD)				CL (X±SD)

	F_P_	F_S_	F_T_	F_dG_	
C−	14.2^d^±1.36	11.2^d^±0.28	6.5^b^±0.53	7.5^d^±0.74	2.3^a^±0.35
C+	7.1^ab^±0.12	3.7^a^±1.17	3.8^a^±1.21	1.5^a^±0.62	2.3^a^±0.34
T1	6.2^a^±0.16	5.2^b^±0.16	3.6^a^±0.16	2.6^b^±0.19	2.6^a^±0.10
T2	7.8^bc^±0.28	5.8^b^±0.53	3.7^a^±0.26	2.7^b^±0.26	2.5^a^±0.10
T3	8.4^c^±0.34	8.4^c^±0.34	4.6^a^±0.34	4.5^c^±1.01	3.4^b^±0.23

Different superscripts in the same column indicate significant differences among treatments (p<0.05). F_P_=Primary follicles; F_S_=Secondary follicles; F_T_=Tertiary follicles; F_dG_=de Graaf’s follicle CL=The corpus luteum, SD=Standard deviation

**Figure-1:**
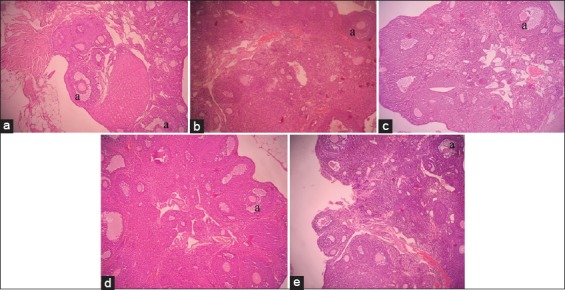
Ovarian histological features in treatments of C−, C+, T1, T2 and T3. (a) Primary follicle; (b) Secondary follicles; (c) Tertiary follicles; (d) de Graaf’s follicle; (e) The corpus luteum.

Data obtained normal distribution than continued with ANOVA test. Statistical test results on the number of the primary follicle, secondary follicle, tertiary follicle, and the de Graaf’s follicle showed significant differences (p<0.05) between C− and C+, T1, T2 and T3. This indicates that the dose of 2 mg/head/day of mycotoxin binders can not inhibit the effects ZEN thus require more doses of mycotoxin binders to be able to inhibit the effects ZEN. The number of CL showed results was not significantly different (p>0.05) between the C− and C+, T1 and T2. This means that the mycotoxin binders cannot reduce the effect of ZEN on the number of CL (Figure-1).

## Discussion

The study of ZEN by Gajecka [8] found that, ZEN can suppress the proliferative activity of follicle cells contained in the ovarian cortex. The decline in follicular cells count was not followed with oocyte maturation process and follicular liquor influx that physiologically fills the antral space in the de Graaf’s follicle before ovulation process. Follicular liquor is identical to endogenous estrogen hormones, and if collected with exogenous estrogen, may lead to hyperestrogenism.

ZEN is metabolized in the liver by the enzyme hydroxysteroid dehydrogenase into two metabolites isomers, the alpha ZEN and beta ZEN. Both of these isomers have resorcylic acid lactone structure that can penetrate the cell membrane of single layer cuboidal epithelial cells constituting the GCs to bind to estrogen receptors (E2) in the cytosol. Complex binding in the form of ZEN E2 receptor complex (ZEA-E2R) will be transferred into the nucleus and bind to estrogen receptors specifically in the nucleus, which may activate mRNA response which is usually played by estrogen receptors (E2) [9,10].

ZEA-E2R, which has enabled mRNA response, may inhibit the mitotic process of granulose cumulus cells on primary follicle. The GCs may develop to form layers of mature GCs and produce some cytokines such as insulin-like growth factor-1 (IGF-1), fibroblastic growth factor family-2 (FGF-2), and stem cell factor (SCF). SCF will initiate oocytes development in primary follicles and stimulates theca cell mitosis. FGF-2 will suppress apoptosis of GCs and help SCF in maturating oocytes. IGF-1 plays an important role in sending impulses to the anterior pituitary to release follicle stimulating hormone (FSH) as an initiator of follicular proliferation. FSH will then be captured by the receptor located in GCs and regulates primary follicle development to become de Graaf’s follicle [11-13].

Effects of high doses ZEN may inhibit GCs in inducing IGF-1, leading to the decrease of FSH production. Superovulatory induction, which uses pregnant mare serum gonadotropin hormone derivatives, may accelerate the development of the follicle and an increased the number of de Graaf’s follicle in the preovulatory stage [14,15].

The results of this study were supported by the opinion of Motea and Berdis [16], Moreira *et al*. [17] who wrote that ZEN metabolites may damage nucleic acid of pre-puberty female canine oocytes. The apoptotic profile is seen in oocytes with TUNEL staining assay.

As a major source of steroid hormones synthesis, cholesterol will be broken down to pregnenolone by the enzyme cytochrome p450 (CYP450) in the inner part of mitochondrial membrane. Pregnenolone is converted to progesterone by the enzyme 3beta-hydroxysteroid dehydrogenase (3beta-HSD) after diffuses toward the endoplasmic reticulum and to become dehydroepiandrosterone (DHEA) by the enzyme CYP450 17beta-hydroxylase (CYP450-17beta). 3beta-HSD and CYP450-17beta will catalyze DHEA and progesterone to androstendion. Androstendion will be catalyzed by the enzyme 17beta-HSD into testosterone in the theca cells and will diffuse to the GCs. Testosterone and estrone in GCs will be transformed into 17beta-estradiol by the enzyme aromatase (CYP450arom). Excessive accumulation of 17beta-estradiol, coupled with the influx of exogenous estrogen, may cause hyperestrogenism [18,19].

Mycotoxin binders can bind mycotoxins that have silicate compound. Silicate structure consists of neosilicate, sorosilicate, inosilicate, cyclosilicate, phyllosilicate, and tectosilicate. The structure will be transformed into hydrated sodium calcium aluminosilicate that is easily metabolized body. Mycotoxin binders are also equipped with a decontaminant material useful in improving the animals’ condition [20,21].

Mycotoxin binders cannot reduce effects caused by ZEN on the number of CL. ZEN only gives effect on GCs up to preovulatory stage. The CL formed during postovulatory stage will be influenced by the amount of luteinizing hormone (LH) surge levels captured by LH receptors on theca cells of the de Graaf’s follicle. Oocytes that have been ovulated from de Graaf’s follicle will cause scars called the corpus rubrum and developed into the CL [2,12].

## Conclusion

The number of follicles and CL more in line with increasing doses of mycotoxin binders. Required more than 2 mg/mouse/day mycotoxin binders to inhibit the effects of ZEN so that its can maintain the number of primary follicle, secondary follicle, tertiary follicle, the de Graaf’s follicle, and the number of CL in the ovary of ZEN-exposed female mice (*M. musculus*). Further studies should be performed on the potential of mycotoxin binders on the physiology and pathology of ovary, fetal placenta, the number of fetuses, testosterone and estrogen levels in mice exposed to ZEN.

## Authors’ Contributions

AS: Research project leader and coordinating research, designed study and analyzed data. ES: Processing of immunohistochemical method and corresponding author. Both authors read and approved the final manuscript.

## References

[1] Hariadi M, Hardjopranjoto S, Wurlina Hermadi H.A, Utomo B, Rimayanti Triana I.N, Ratnani H (2011). Ilmu Kemajiran Padaternak. Cetakan 1.

[2] Hafez E.S.E (2000). Folliculogenesis, egg maturation, and ovulation. Reproduction in Farm Animal.

[3] Kumar V, Cotran R.S, Robins S (2005). Basic Pathology.

[4] Guerrero A.D, Schmitz I, Chen M, Wang J (2012). Promotion of caspase activation by caspase 9 mediated feedback amplification of mitochondrial damage. J. Clin. Cell. Immunol.

[5] Avantaggiato G, Solfrizzo M, Visconti A (2005). Recent advances on the use of adsorbent materials for detoxification of Fusarium mycotoxins. Food Addit. Contam.

[6] Döll S, Dänicke S, Valenta H, Flachowsky G (2004). *In vitro* studies on the evaluation of mycotoxin detoxifying agents for their efficacy on deoxynivalenol and zearalenone. Arch. Anim. Nutr.

[7] Sinuhaji I, Siregar B, Lisnawati (2013). Revised: Ekspresi p16^INK4A^ on cervic carcinoma young age. Departement of Anatomy Patology. Medicine Faculty. Indonesia University. Jakarta. J. Indon. Med. Assoc.

[8] Gajecka M (2013). The effect of experimental low zearalenone intoxication on ovarian follicles in pre-pubertal bitches. Pol. J. Vet. Sci.

[9] Malekinejad H, Mass-Bakker R.F, Fink-Gremmels J (2005). Bioactivation of zearalenone by porcine hepatic biotransformation. Vet. Res.

[10] Frizzell C, Ndossi D, Verhaegen S, Dahl E, Eriksen G, Srrlie M, Ropstad E, Muller M, Elliott C.T, Connolly L (2011). Endocrine disrupting effects of zearalenone, alpha-and beta-zearalenol at the level of nuclear receptor binding and steroidogenesis. Toxicol. Lett.

[11] Tiemann U, Viergutz T, Jonas L, Schneider F (2003). Influence of the mycotoxins α-and β-zearalenol and deoxynivalenol on the cell cycle of cultured porcine endometrial cells. Reprod. Toxicol.

[12] Johnson M.H (2007). Ovarian function in the adult. Essential Reproduction.

[13] Ranzenigo G, Caloni F, Cremonesi F, Aad P.Y, Spicer L.J (2008). Effects of Fusarium mycotoxin on steroid production by porcine granulose cells. Anim. Reprod. Sci.

[14] Yusuf T.L, Toelihere M.R, Supriatna I, Arifiantini L (1993). Use of Various Doses Pregnant Mare Serum Gonadotropin (PMSG) For Activities Superovulation and Transfer FH.

[15] Ndossi D.G, Frizzell C, Tremoen N.H, Faeste C.K, Verhaegen S, Dahl E, Eriksen G.S, Sorlie M, Connolly L, Ropstad E (2012). An *in vitro* investigation of endocrine disrupting effects of trichothecenes deoxynivalenol (DON), T-2 and HT-2 toxin. Toxicol. Lett.

[16] Motea E.A, Berdis A.J (2010). Terminal deoxynucleotidyl transferase: The story of a misguided DNA polymerase. Biochem. Biophys. Acta.

[17] Moreira P.I, Custódio J.B, Nunes E, Oliveira P.J, Moreno A, Seica R, Oliveira C.R, Santos M.S (2011). Mitochondria from distinct tissues are differently affected by 17β-es-tradiol and tamoxifen. J. Steroid Biochem. Mol. Biol.

[18] Boron F.W, Boulpaep L.E (2003). Medical Physiology: A Cellular and Molecular Approach.

[19] Tsuchiya Y, Nakajima M, Yokoi T (2005). Cytochrome p450-mediated metabolism of estrogens and its regulation in human. Cancer Lett.

[20] Bingham A.K, Phillips T.D, Bauer J.E (2003). Potential for dietary protection against the effects of aflatoxins in animals. J. Am. Vet. Med. Assoc.

[21] Whitlow L.W, Hagler W.M (2005). Mycotoxins in feeds. Feedstuffs.

